# Percutaneous transhepatic vascular embolization for a mesenteric arteriovenous malformation leading to decreased portal pressure in a patient with alcoholic liver cirrhosis: A case report

**DOI:** 10.1016/j.radcr.2025.03.037

**Published:** 2025-04-05

**Authors:** Ken Shigeyama, Yasuyuki Ono, Shuji Kariya, Miyuki Nakatani, Takuji Maruyama, Yuki Tanaka, Takayuki Matsumoto, Hiroyuki Fujita, Noboru Tanigawa

**Affiliations:** a Department of Radiology, Pref Osaka Saiseikai Izuo Hospital, 3-4-5 Kitamura, Taisho-ku, Osaka City, Osaka 5510032, Japan; b Department of Radiology, Kansai Medical University, 2-5-1 Shinmachi, Hirakata, Osaka 5731010, Japan; c Division of Gastroenterology, Department of Internal Medicine, Pref Osaka Saiseikai Izuo Hospital, 3-4-5 Kitamura, Taisho-ku, Osaka City, Osaka 5510032, Japan

**Keywords:** Mesenteric arteriovenous malformation, Portal hypertension, Interventional radiology, Transcatheter embolization, Case report

## Abstract

Arteriovenous malformations in the abdominal region are rare, and they are known to occasionally cause portal hypertension. A 66-year-old man with a chief complaint of fatigue and blood tests showing hepatic dysfunction was seen for a more detailed examination. Abdominal contrast-enhanced computed tomography showed an anastomosis of the ileal artery and ileal vein via a nidus within the mesentery. In addition to alcoholic cirrhosis from a history of high alcohol intake, the patient was diagnosed with portal hypertension from increased portal pressure due to an arteriovenous malformation in the mesentery. Vascular embolization with a percutaneous transhepatic approach was performed for the mesenteric arteriovenous malformation. The ileal vein, which was the dominant outflow vein, was embolized, and the blood flow in the arteriovenous malformation disappeared. A decrease in portal pressure of 29% was confirmed. There were no complications from the embolization. In cases of mesenteric arteriovenous malformations that contribute to portal hypertension, treatment of the malformations can be expected to decrease portal pressure. Compared with surgical intestinal resection, endovascular treatment that can be done with low invasiveness is thought to be a possible option.

## Introduction

Arteriovenous malformations (AVMs) occur most frequently in the head and neck region, followed by the limbs, trunk, and viscera [1]. In the intestinal tract, they are considered to occur most frequently in the cecum and right colon, with development in the ileum accounting for 5.5% of cases [2]. A mesenteric AVM sometimes produces portal hypertension (PH), and PH is reportedly seen in 50% of congenital inferior mesenteric arteriovenous fistulae [3].

A case in which portal pressure was decreased by percutaneous transhepatic vascular embolization of an idiopathic mesenteric AVM thought to have contributed to PH is reported.

## Case

A 66-year-old man presented with a chief complaint of fatigue. His medical history included fatty liver, hypertension, hyperuricemia, and chronic gastritis. Blood tests showed hepatic dysfunction, and a more detailed workup was performed. He regularly consumed about 100-180 g per day of alcohol.

No abnormalities were seen on physical examination. The results of blood tests are shown in Table 1. He tested negative for hepatitis B, hepatitis C virus, and antinuclear and antimitochondrial antibodies.

**Table 1:** Laboratory test results.

Test items	Results	Units
White blood cell	7030	/μL
Hemoglobin	16.2	g/dL
Platelets	141,000	/μL
PT activity	92.1	%
PT-INR	1.04	
Aspartate aminotransferase	55	U/L
Alanine aminotransferase	58	U/L
γ-glutamyl transpeptidase	357	U/L
Total bilirubin	1.6	mg/dL
Albumin	4.1	g/dL
Triglycerides	142	mg/dL
Low-density lipoprotein cholesterol	141	mg/dL
Sodium	139	mEq/L
Pottassium	4.4	mEq/L
Chloride	105	mEq/L
Iron	156	μg/dL
Copper	112	μg/dL
Creatinine	0.78	mg/dL
C-reactive protein	0.32	mg/dL

On abdominal ultrasound, coarsened echotexture, surface nodularity, and hypertrophy of the caudate and lateral segment with volume loss of the right lobe were seen in the liver, suggesting cirrhosis. Splenomegaly was also seen. There was no ascites.

**Fig. 1:**
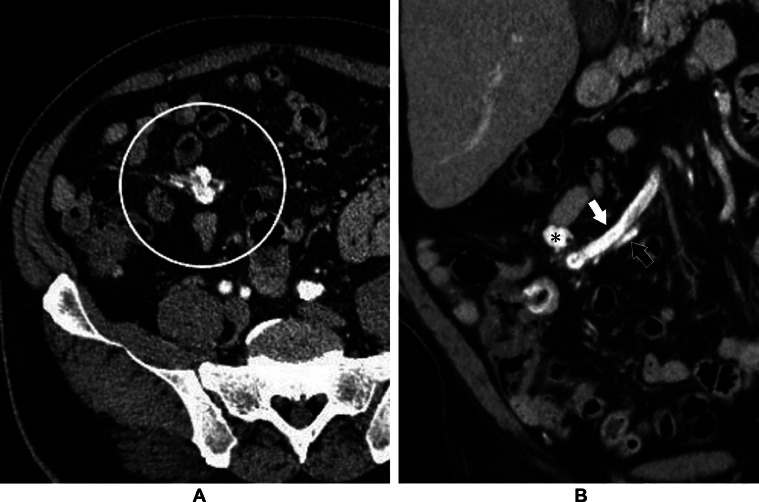
Abdominal contrast-enhanced computed tomography, early phase. (A) Axial image. An irregular, dilated vascular structure is visible in the mesentery (white circle). (B) Coronal image. The ileal artery (black arrow) connects with the dilated ileal vein (white arrow) via the dominant outflow vein (asterisk). Contrast enhancement is visible in the ileal vein at about the same level as in the ileal artery, leading to the diagnosis of an arteriovenous malformation.

On abdominal contrast-enhanced computed tomography (CT; Incisive CT, Philips Japan, Tokyo, Japan), blunting of the liver edge and splenomegaly were confirmed. An anastomosis of the ileal artery and ileal vein via a nidus within the mesentery was also identified (Fig. 1A). In the early phase of contrast-enhanced CT, there was contrast enhancement in the ileal vein at the same level as the artery (Fig. 1B). A mesenteric AVM was diagnosed based on these findings.

On upper gastrointestinal endoscopy, varices equivalent to F1–2 were seen sporadically in the esophagus from 35 cm from the incisors through the gastroesophageal junction.

**Fig. 2:**
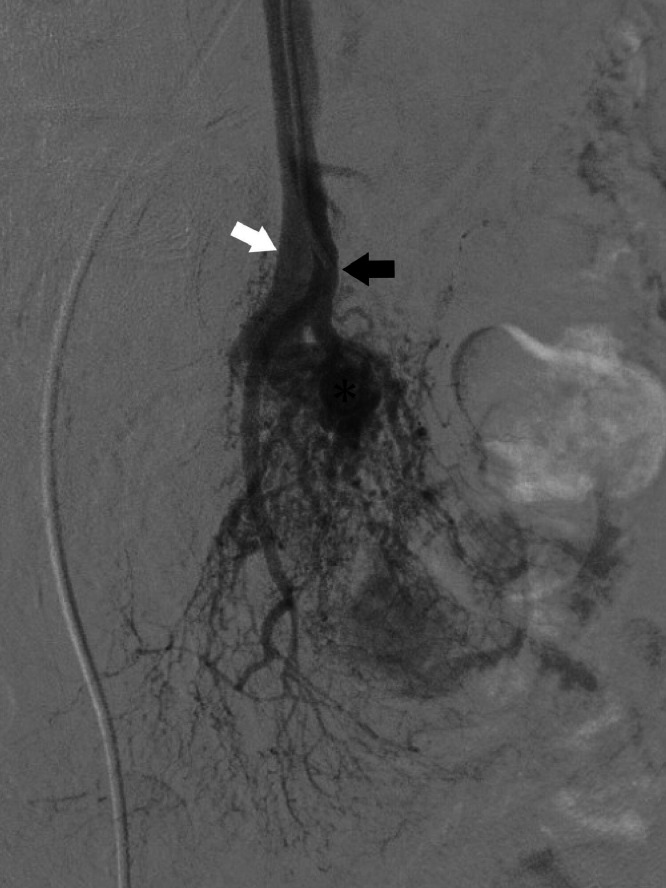
Digital subtraction angiography with contrast enhancement from the ileal artery. Multiple inflow channels from the ileal arteries (black arrows) and the dominant outflow vein (asterisk) are shown, and outflow to one dilated ileal vein (white arrow) is visible.

An angiographic examination was performed to ascertain the hemodynamics of the AVM (Azurion, Philips Japan). Arteriography of the superior mesenteric artery (SMA) showed ileal artery dilation. On ileal arteriography, a single dilated ileal vein, which was the dominant outflow vein (DOV) via the nidus, was confirmed (Fig. 2). The inflow route consisted of multiple small diameter arteries, whereas the outflow route was a single ileal vein. The shunt flow from the artery was fast, and there was thought to be a pressure load on the portal vein.

In addition to alcoholic cirrhosis from a history of high alcohol consumption, PH was diagnosed from an increased portal pressure load due to the AVM that occurred in the mesentery. In addition to abstinence from alcohol, treatment of the AVM was considered to reduce the risk of future worsening of varices and liver damage. Embolization with a percutaneous transhepatic approach was performed for the mesenteric AVM. The right hepatic lobe was punctured using an ultrasound-guided, 22-gauge, 20-cm Chiba needle (Chiba biopsy needle, Angiotech, Gainesville, FL, USA). A 5-Fr, 25-cm-long sheath introducer (Super Sheath, Medikit, Miyazaki, Japan) was placed using a 0.018-inch wire (Radifocus, Terumo, Tokyo, Japan) after confirming the portal vein. A 5.2-Fr, cobra-shaped, 9-mm-diameter occlusion balloon catheter with an open tip (Selecon MP Catheter II, Terumo Clinical Supply, Gifu, Japan) was placed in the DOV of the peripheral ileal vein. An angled shape-2 marked microcatheter (Lighthouse, Piolax, Kanagawa, Japan) was inserted as a catheter for embolization. A 5-Fr, cobra-shaped catheter (C2, Medikit) was placed from the right femoral artery into the SMA as the contrast route from the artery.

When the portal pressure was measured prior to embolization, the mean pressure of the main trunk of the portal vein was 28 mmHg.

**Fig. 3:**
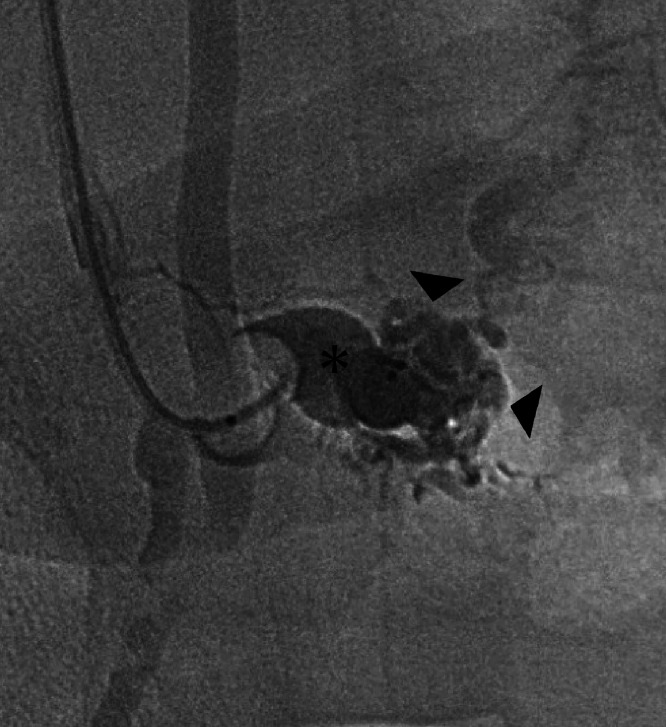
Digital radiography image with balloon occlusion of the dominant outflow vein, a blood drainage channel, from a percutaneous transhepatic route, and retrograde contrast. Multiple nidi (black arrowheads) via the dominant outflow vein (asterisk) are confirmed, leading to the diagnosis of an arteriovenous malformation of Type II in the Cho classification.

**Fig. 4:**
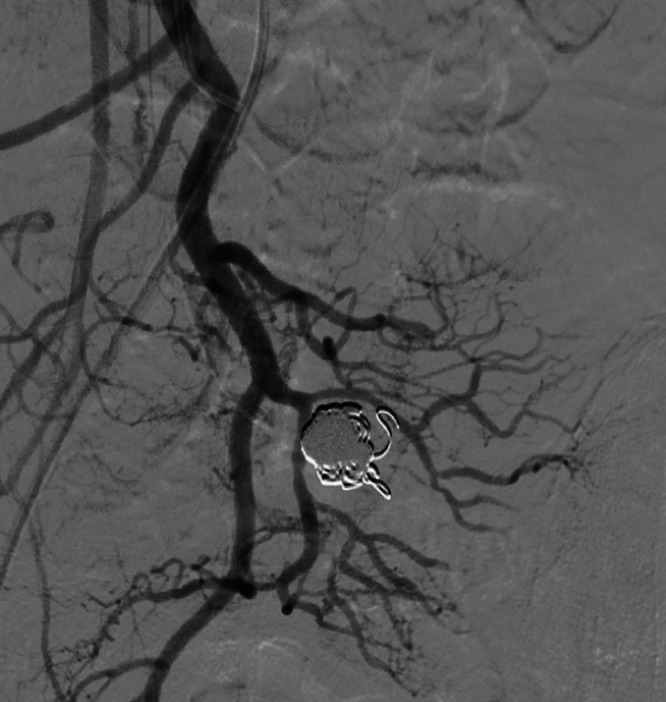
Digital subtraction angiography from the superior mesenteric artery embolization. After embolization of the dominant outflow vein and nidus with 5% ethanolamine oleate with iopamidol and coils, blood flow through the arteriovenous malformation is confirmed to have disappeared.

Balloon occlusion of the DOV, the outflow channel, was performed, and a nidus was confirmed with retrograde contrast (Fig. 3). Stagnation of the blood flow from balloon occlusion was also seen. Flow control from the arterial side was considered, but embolization was judged to be possible with DOV flow control alone. The DOV was embolized using 5% ethanolamine oleate with iopamidol 3 mL, 4 to 8-mm-diameter fibered coils (EMBOLD, Boston Scientific, Tokyo, Japan), and 6-mm-diameter metallic coils (Target XXL 360, Stryker Japan, Tokyo, Japan). On SMA arteriography after embolization, the blood flow of the AVM was confirmed to have disappeared (Fig. 4). The mean pressure of the main trunk of the portal vein was confirmed to have decreased to 20 mmHg with embolization. The intrahepatic puncture route was embolized using 0.4 mL of a 1:1 mixed solution of n‑butyl‑2-cyanoacrylate and lipiodol. There were no complications from the embolization.

**Fig. 5:**
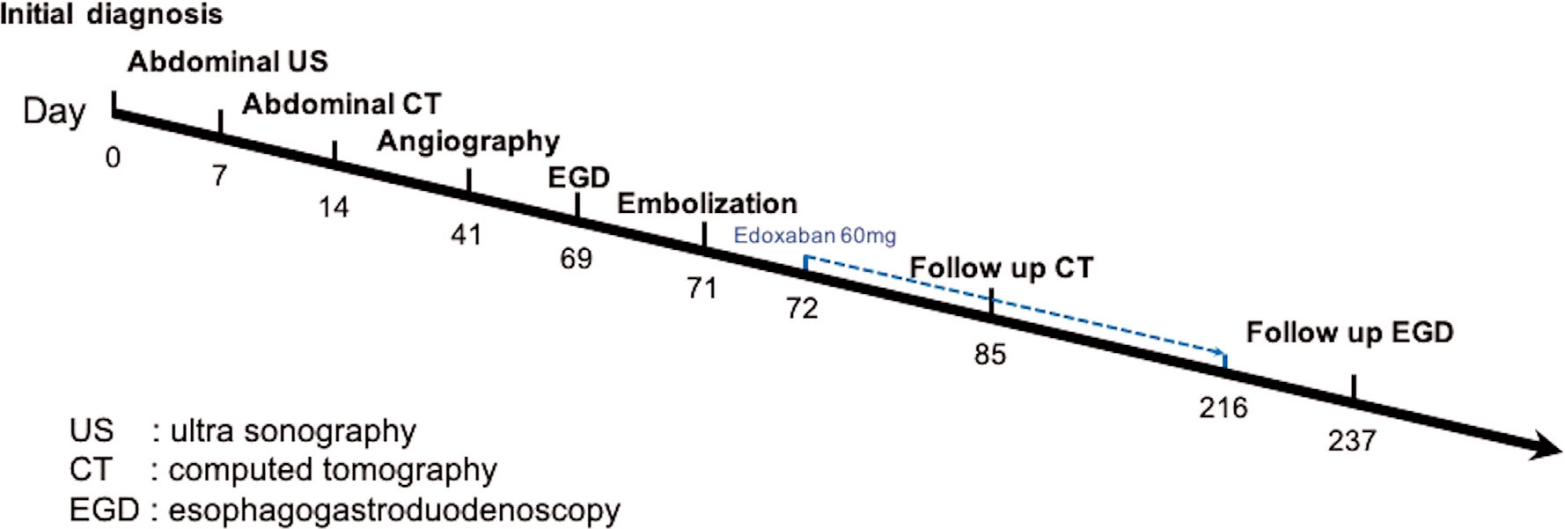
Time line summarizing key events.

Fig. 5 shows the timeline from the initial diagnosis through follow-up. Oral administration of edoxaban 60 mg was started to prevent portal vein thrombosis with the decrease in portal vein blood flow after embolization. Abdominal contrast-enhanced CT was performed on the 16th day after embolization. Shrinkage of the dilated ileal vein and disappearance of the shunt blood flow seen in the early phase were confirmed. No thrombus was seen in the portal vein. On upper gastrointestinal endoscopy performed 6 months after embolization, there was no apparent worsening of the esophageal varices. Furthermore, no worsening of liver function tests was observed.

## Discussion

In this case, a mesenteric AVM was seen against a background of alcoholic cirrhosis. On angiography, there was much blood flow through the AVM to the portal vein, and this AVM was also thought to be one cause of the PH.

In PH, it has been reported that the complication rate and mortality rate are significantly reduced with decreased pressure of 20% or more from baseline in the hepatic venous pressure gradient used as a substitute for portal pressure [[4], [5], [6]]. One case was also reported in which portal pressure actually decreased from 46 mmHg to 26 mmHg with endovascular treatment of an AVM in which PH was seen [7].

In the present patient as well, portal trunk pressure decreased from 28 mmHg to 20 mmHg with vascular embolization of the AVM, and a decrease in portal pressure of 29% from baseline was obtained. The risk of complications associated with PH seems to have been decreased.

In the present case, habitual alcohol intake was considered to be the main factor contributing to liver dysfunction and portal hypertension, but due to the patient's psychosocial background, he continued to consume alcohol despite being advised to abstain from alcohol intake. Therefore, further treatment intervention was considered necessary. No worsening of liver damage or worsening of varicose veins was observed though alcohol intake continued after treatment.

The classifications of Cho are generally used in classifying AVMs. In this patient, multiple inflow arteries and one DOV were observed on angiography, and this was thought to correspond to Type II. Good treatment results are reported with direct puncture and a transvenous approach for Type II AVMs [8].

In treating AVMs in the mesenteric region, surgery is often selected due to the risks of mesenteric ischemia and recurrence with vascular embolism [9,10]. In the present patient, there was concern about the risk of mesenteric ischemia with arterial embolism. However, there was thought to be little risk of mesenteric ischemia with embolization using an approach from the venous side. In AVMs of the mesentery, embolization from the arterial side is reported because, with a venous approach, it proceeds via the portal vein, and it is difficult to reach the periphery, but there are few reports of embolization of an AVM from the venous side [[11], [12], [13]]. On angiography, since the AVM was a Type II with a single DOV, and the vascular route to the DOV was straight, it was determined that catheter insertion to the DOV and embolization under flow control was possible. Therefore, in the present case, a percutaneous transhepatic route was used to embolize the ileal vein, the DOV. Embolization of the AVM was performed without intestinal ischemia or complications.

Although no recurrence of AVM or portal vein thrombus was observed 6 months after embolization, regular follow-up with contrast-enhanced CT is necessary because of the risk of recurrent AVM if DOV embolization is inadequate and thrombus forms due to reduced portal vein blood flow. When a mesenteric AVM that contributes to PH is seen in patients with a background of cirrhosis, decreased portal pressure can be expected with treatment, and compared with surgical intestinal resection, it is thought that low-invasive endovascular treatment can be an option.

## Conclusion

In the present case, percutaneous transhepatic vascular embolization for an AVM of the mesentery that contributed to PH was performed, and portal pressure decreased 29%.

## Patient consent

Written informed consent for the publication of this case report was obtained from the patient.
